# Supplementary material for: Low oxygen environment alters transcripts related to energy metabolism without altering the pluripotency core of bovine embryonic stem cells
Source: Biol Open. 2026 May 11;15(5):bio062352. doi: 10.1242/bio.062352 (PMC13225197; doi:10.1242/bio.062352)
Supplement: Supplementary information [file biolopen-15-062352-s1.pdf]

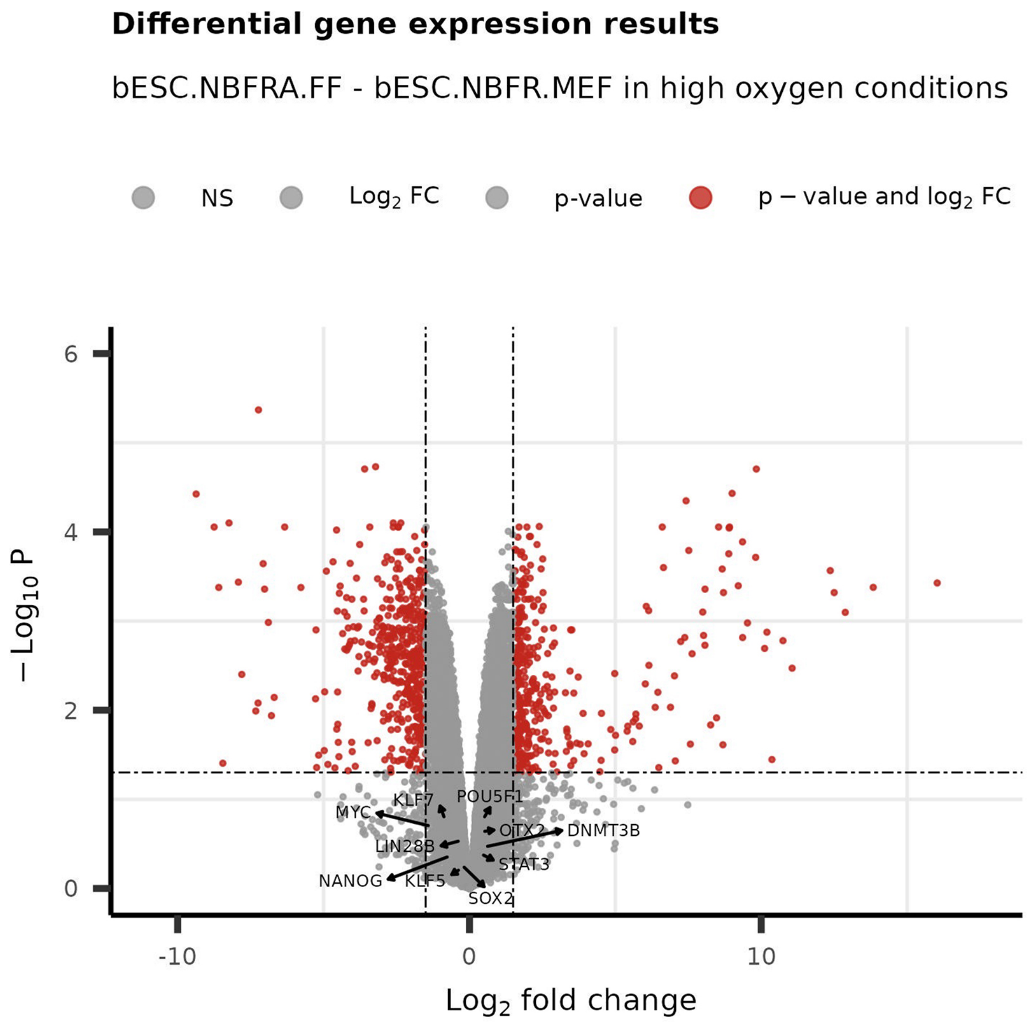

Fig. S1. Volcano plot showing genes associated with the core of pluripotency not included in the significantly downregulated and upregulated genes between bESCs in feeder-free vs on MEF culture conditions in high oxygen.

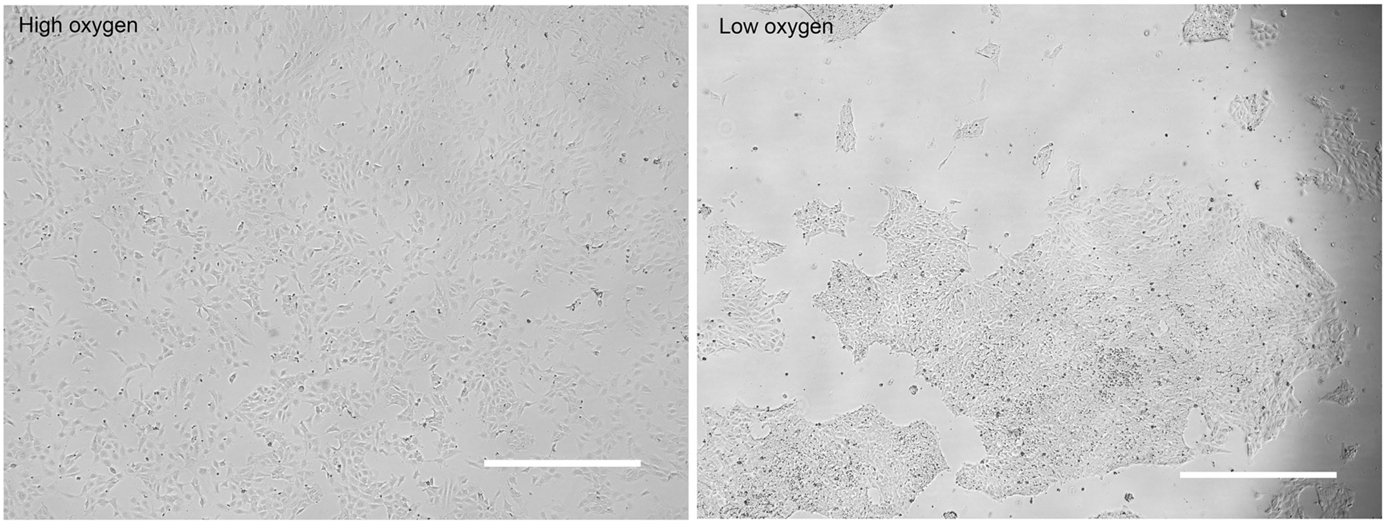

Fig. S2. Representative image of bESCs in feeder-free conditions cultured in high or low oxygen, showing highly-packed colonies on low oxygen but not in high oxygen conditions. Scale bars: 750 μm.

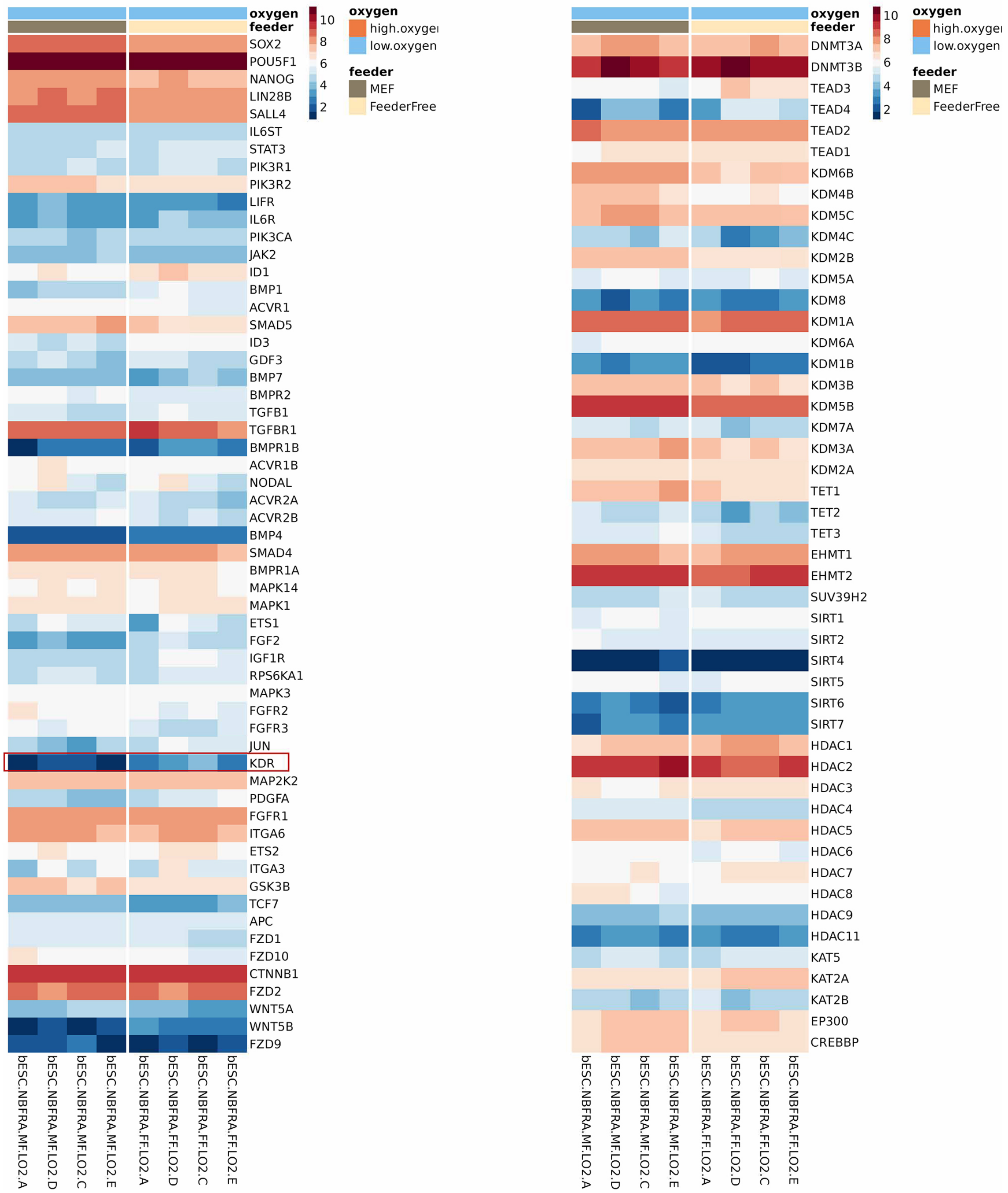

Fig. S3. Heatmap of genes associated with pluripotent core, JAK/STAT3, TGF-β/SMADs, FGF/ERK, and WNT/β-Catenin pathways and epigenetic markers for bESCs in low oxygen cultured in MEF or feeder-free conditions.

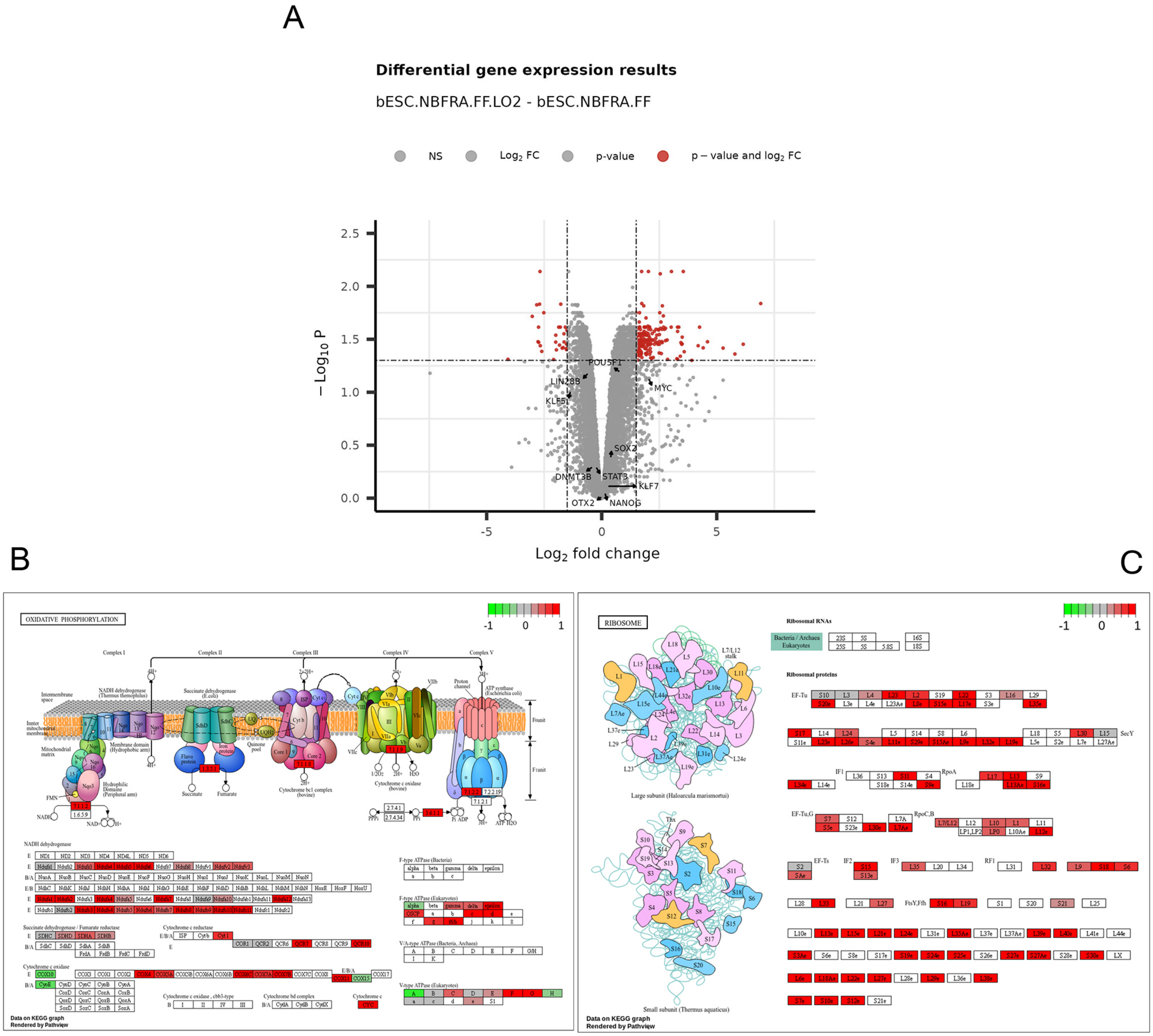

Fig. S4. Volcano plot showing genes associated with the core of pluripotency not included in the significantly downregulated or upregulated genes between bESCs low oxygen vs high oxygen in feeder-free conditions. (B) Pathview plot showing upregulated genes in low oxygen on the oxidative phosphorylation cascade. (C) Pathview plot showing upregulated genes in low oxygen on the ribosomal cascade. B-C genes highlighted in red were upregulated in low oxygen environment.

Biology Open • Supplementary information

Table S1. Gene Set Enrichment Analysis. Enriched pathways based on differential gene expression of bovine ESC in high oxygen cultured in feeder-free compared with culture in feeder (MEF) conditions.

Enriched pathways downregulated in FeederFree vs MEFs

ID	Description	setSize	enrichmentScore	NES	pvalue	p.adjust	qvalue	rank	leading_edge	core_enrichment
GO:0010646	regulation of cell communication	9	-0.726	-2.026	0.001	0.047	0.041	51	tags=67%, list=13%, signal=59%	408005/777788/508794/618405/522006/282261
GO:0023051	regulation of signaling	9	-0.726	-2.026	0.001	0.047	0.041	51	tags=67%, list=13%, signal=59%	408005/777788/508794/618405/522006/282261

Enriched pathways upregulated in FeederFree vs MEFs

ID	Description	setSize	enrichmentScore	NES	pvalue	p.adjust	qvalue	rank	leading_edge	core_enrichment
GO:0060485	mesenchyme development	5	0.940	1.922	0.000	0.001	0.001	12	tags=80%, list=3%, signal=79%	282187/515610/521378/538602
GO:0061448	connective tissue development	5	0.895	1.831	0.000	0.007	0.004	12	tags=60%, list=3%, signal=59%	282187/515610/538602
GO:0048705	skeletal system morphogenesis	5	0.895	1.830	0.000	0.007	0.004	12	tags=60%, list=3%, signal=59%	282187/521378/538602
GO:0060349	bone morphogenesis	5	0.895	1.830	0.000	0.007	0.004	12	tags=60%, list=3%, signal=59%	282187/521378/538602
GO:0065008	regulation of biological quality	15	0.736	1.958	0.000	0.009	0.005	36	tags=47%, list=9%, signal=44%	280794/515610/538602/282478/281793/286810/282004
GO:0048646	anatomical structure formation involved in morphogenesis	16	0.712	1.911	0.000	0.009	0.006	25	tags=38%, list=6%, signal=37%	282187/280794/515610/521378/538602/281793
GO:0032502	developmental process	35	0.602	1.841	0.000	0.009	0.006	55	tags=34%, list=13%, signal=32%	282187/280794/515610/521378/538602/281793/515181/286810/282004/338319/327708/281572
GO:0003008	system process	9	0.801	1.911	0.000	0.010	0.006	53	tags=67%, list=13%, signal=59%	282187/515610/538602/281793/282004/532713
GO:0042221	response to chemical	22	0.631	1.803	0.002	0.031	0.018	56	tags=41%, list=14%, signal=37%	282187/521378/538602/281793/286810/404181/327708/281572/504742
GO:0097435	supramolecular fiber organization	10	0.734	1.804	0.002	0.032	0.019	12	tags=30%, list=3%, signal=30%	282187/515610/538602

Biology Open • Supplementary information

Table S2. Over-Representation Analysis (ORA). Enriched pathways based on differential gene expression of bovine ESC in feeder-free culture under low or high oxygen conditions.

- **Enriched pathways downregulated in low oxygen vs high oxygen**

ID	Description	GeneRatio	BgRatio	pvalue	p.adjust	qvalue	geneID	Count
GO:0071417	cellular response to organonitrogen compound	5/9	121/4435	1.61E-06	0.00066288	0.0004544	AGTR1/COL1A1/CHRM3/ATP2B4/VIM	5
GO:1901699	cellular response to nitrogen compound	5/9	134/4435	2.67E-06	0.00066288	0.0004544	AGTR1/COL1A1/CHRM3/ATP2B4/VIM	5
GO:0010243	response to organonitrogen compound	5/9	180/4435	1.15E-05	0.00190193	0.00130374	AGTR1/COL1A1/CHRM3/ATP2B4/VIM	5
GO:1901701	cellular response to oxygen-containing compound	5/9	231/4435	3.90E-05	0.00386533	0.00264962	AGTR1/COL1A1/CHRM3/ATP2B4/VIM	5
GO:0071495	cellular response to endogenous stimulus	5/9	254/4435	6.18E-05	0.00510506	0.00349944	AGTR1/COL1A1/CHRM3/ATP2B4/VIM	5
GO:0048762	mesenchymal cell differentiation	3/9	49/4435	0.00010164	0.0072022	0.00493699	CDH2/COL1A1/HAS2	3
GO:1905144	response to acetylcholine	2/9	10/4435	0.00016338	0.00749329	0.00513653	CHRM3/ATP2B4	2
GO:1901700	response to oxygen-containing compound	5/9	312/4435	0.00016618	0.00749329	0.00513653	AGTR1/COL1A1/CHRM3/ATP2B4/VIM	5
GO:0032964	collagen biosynthetic process	2/9	11/4435	0.00019948	0.00824497	0.00565179	COL1A1/VIM	2
GO:0003008	system process	5/9	335/4435	0.00023337	0.00890405	0.00610358	AGTR1/COL1A1/CHRM3/ATP2B4/HAS2	5
GO:0071310	cellular response to organic substance	5/9	364/4435	0.00034618	0.01226468	0.00840724	AGTR1/COL1A1/CHRM3/ATP2B4/VIM	5
GO:0001101	response to acid chemical	2/9	22/4435	0.00082814	0.02738381	0.01877116	COL1A1/ATP2B4	2
GO:0032963	collagen metabolic process	2/9	23/4435	0.00090605	0.02808768	0.01925365	COL1A1/VIM	2
GO:0044057	regulation of system process	3/9	106/4435	0.00100397	0.02929214	0.02007929	AGTR1/CHRM3/ATP2B4	3
GO:0016477	cell migration	4/9	265/4435	0.001236185	0.03339551	0.022892083	AGTR1/COL1A1/IGFBP6/ATP2B4	4

- **Enriched pathways upregulated in low oxygen vs high oxygen**

ID	Description	GeneRatio	BgRatio	pvalue	p.adjust	qvalue	geneID	Count
GO:0006412	translation	25/75	247/4435	5.87E-14	3.91E-11	3.81E-11	GRB7/RPS28/RPS29/RPL35/RPL24/RPS17/RPS12/RPL38/RPL27/RPS15A/RPLP2/RPL26/RPS19/RPS7/RPS18/RPS11/RPL14/RPL37A/RPLP1/RPL13A/RPS6/RPS21/SHFL/RPS24/RPL36A	25
GO:0043043	peptide biosynthetic process	25/75	254/4435	1.13E-13	3.91E-11	3.81E-11	GRB7/RPS28/RPS29/RPL35/RPL24/RPS17/RPS12/RPL38/RPL27/RPS15A/RPLP2/RPL26/RPS19/RPS7/RPS18/RPS11/RPL14/RPL37A/RPLP1/RPL13A/RPS6/RPS21/SHFL/RPS24/RPL36A	25
GO:0043604	amide biosynthetic process	26/75	279/4435	1.17E-13	3.91E-11	3.81E-11	GRB7/RPS28/RPS29/SNCA/RPL35/RPL24/RPS17/RPS12/RPL38/RPL27/RPS15A/RPLP2/RPL26/RPS19/RPS7/RPS18/RPS11/RPL14/RPL37A/RPLP1/RPL13A/RPS6/RPS21/SHFL/RPS24/RPL36A	26
GO:1901566	organonitrogen compound biosynthetic process	30/75	476/4435	2.71E-11	4.53E-09	4.41E-09	GRB7/AK4/SLC44A4/ATP5ME/RPS28/RPS29/SNCA/RPL35/RPL24/RPS17/RPS12/RPL38/RPL27/RPS15A/RPLP2/RPL26/RPS19/RPS7/RPS18/RPS11/RPL14/RPL37A/ATP5IF1/RPLP1/RPL13A/RPS6/RPS21/SHFL/RPS24/RPL36A	30
GO:0042274	ribosomal small subunit biogenesis	9/75	38/4435	7.62E-09	1.09E-06	1.06E-06	RPS28/RPS17/RPS12/RPL38/RPS15A/RPS19/RPS11/RPS6/RPS24	9
GO:0007005	mitochondrion organization	12/75	184/4435	4.79E-05	0.00415006	0.00404215	ACAA2/BNIP3/FAM162A/NDUFB3/SNCA/TOMM7/NDUFC1/NDUFA1/NDUFB1/NDUFA3/NDUFB4/MGARP	12
GO:0010257	NADH dehydrogenase complex assembly	6/75	41/4435	5.37E-05	0.00415006	0.00404215	NDUFB3/NDUFC1/NDUFA1/NDUFB1/NDUFA3/NDUFB4	6
GO:0032981	mitochondrial respiratory chain complex I assembly	6/75	41/4435	5.37E-05	0.00415006	0.00404215	NDUFB3/NDUFC1/NDUFA1/NDUFB1/NDUFA3/NDUFB4	6
GO:0001666	response to hypoxia	6/75	47/4435	0.00011842	0.00849214	0.00827133	AK4/DDIT4/FAM162A/VEGFA/EGR1/MGARP	6
GO:0071456	cellular response to hypoxia	4/75	22/4435	0.00043816	0.02443966	0.0238042	AK4/FAM162A/VEGFA/MGARP	4

Table S3. Antibodies used for immunolocalization and western blotting analysis. Primary antibodies were reconstituted to the concentration specified by manufacturer instructions.

Primary Antibodies	Brand	Catalog number	Method	Concentration
Goat anti-POU5F1	Novus/R&D	AF1759	IF	1:100
Rabbit anti-SOX2	Biogenex	EP103	IF	1:300
Rabbit anti-NANOG	Peprotech	500-P236	IF	1:250
Goat anti-POU5F1	Novus/R&D	AF1759	WB	1:400
Rabbit anti-Histone 3	Abcam	Ab1791	WB	1:5,000

Secondary Antibodies	Brand	Catalog number	Method	Concentration
Donkey anti-goat Alexa Fluor 488	Invitrogen	A11055	IF	1:500
Donkey anti-rabbit Alexa Fluor 647	Invitrogen	A31573	IF	1:500
Peroxidase AffiniPure Donkey Anti-Goat secondary antibody	Jackson ImmunoResearch	705-035-003	WB	1:5,000
Peroxidase Donkey Anti-Rabbit secondary antibody	Jackson ImmunoResearch	711-035-152	WB	1:20,000

Biology Open • Supplementary information
